# Highly diverse RNA viruses and phage sequences concealed within birds

**DOI:** 10.1128/spectrum.00802-24

**Published:** 2024-06-11

**Authors:** Xiang Lu, Likai Ji, Haoning Wang, Qing Zhang, Xiaochun Wang, Yuwei Liu, Quan Shen, Shixing Yang, Xiao Ma, Wen Zhang, Tongling Shan

**Affiliations:** 1 Department of Laboratory Medicine, School of Medicine, Jiangsu University, Zhenjiang, Jiangsu, China; 2 School of Geography and Tourism, Harbin University, Harbin, Heilongjiang, China; 3 Qinghai Institute of Endemic Disease Prevention and Control, Xining, Qinghai, China; 4 Shanghai Veterinary Research Institute, Chinese Academy of Agricultural Sciences, Shanghai, China; Changchun Veterinary Research Institute, Changchun, China

**Keywords:** diversity, RNA virome, metagenomics, birds, phage

## Abstract

The diversity of birds in most parts of the world is very high, and thus, they may carry different types of highly differentiated and unknown viruses. Thanks to advanced sequencing technologies, studies on the diversity of bird-associated viruses have increased over the past few years. In this study, a large-scale viral metagenomics survey was performed on cloacal swabs of 2,990 birds from nine provinces of the Chinese mainland. To detect undescribed RNA viruses in birds, more than 1,800 sequences sharing relatively low (<60%) amino acid sequence identity with the best match in the GenBank database were screened. Potentially novel viruses related to vertebrates have been identified, and several potential recombination signals were found. Additionally, hundreds of RNA viral sequences related to plants, fungi, and insects were detected, including previously unknown viruses. Furthermore, we investigated the novelty, functionality, and classification of the phages examined in this study. These viruses occupied topological positions on the evolutionary trees to a certain extent and might form novel putative families, genera, or species, thus providing information to fill the phylogenetic gaps of related viruses. These findings provided new insights into bird-associated viruses, but the interactions among these viruses remain unknown and require further investigation.

**IMPORTANCE:**

Studying the diversity of RNA viruses in birds and mammals is crucial due to their potential impact on human health and the global ecosystem. Many RNA viruses, such as influenza and coronaviruses, have been shown to cross the species barrier and cause zoonotic diseases. In this metagenomics study involving 2,990 birds from at least 82 species, we identified over 1,800 RNA sequences with distant relationships to known viruses, some of which are rare in birds. The study highlights the scope and diversity of RNA viruses in birds, providing data to predict disease risks and monitor potential viral threats to wildlife, livestock, and human health. This information can aid in the development of strategies for disease prevention and control.

## INTRODUCTION

Viruses are extremely abundant and highly diverse, with an estimated population size of 10^31^ (1, 2). In recent decades, rapid advancements in metagenomics greatly promoted the field of virology and allowed researchers to detect viruses in various objects in the environment, thus increasing information on the diversity of viruses and potentially highly prevalent viruses (3). Birds serve as important reservoirs of pathogenic viruses, posing a significant threat to both human and poultry health (4). Like viruses, birds are highly diverse and ubiquitous in various environments around the world (5, 6). Many species undertake long-distance flights, cross geographic boundaries, and migrate between continents to adapt to seasonal changes (7). For example, mallard (*Anas platyrhynchos*) and greylag goose (*Anser anser*) migrate to China in winter from Alaska, eastern Mongolia, eastern Siberia, and the Russian far east (8). The viruses carried by birds have the potential to undergo mutation and recombination, giving rise to novel viruses that may trigger epidemics in both animals and humans (9). Certain pathogens can be transmitted to birds through untreated sewage and garbage, where they may reproduce and multiply within the avian digestive tract. Subsequently, these pathogens can contaminate commercial poultry farms through fecal matter, leading to transmission to humans (10–12). Besides the avian influenza virus, arboviruses, the *Japanese encephalitis virus*, and the *West Nile virus* can also cause severe zoonotic diseases (4, 13, 14). Therefore, large-scale monitoring of viral infections in birds might help prevent and quickly respond to possible outbreaks in humans or domestic animals.

Due to their inherent instability, RNA viruses have a tendency to undergo recombination within the host organism, facilitating easy transmission to other hosts (15). RNA viruses are also associated with many emerging diseases. From 1990 to 2010, 94% of zoonotic diseases were reported to be caused by RNA viruses (16). Birds act as a reservoir of highly diverse RNA viruses (5), such as astrovirus (17) and picornavirus (18), capable of infecting other vertebrates. Additionally, abundant novel RNA viruses were discovered in different avian hosts, indicating that the viral diversity of avian hosts is not well-known (5, 19–21).

It is increasingly evident that viruses play a crucial role in shaping the health of hosts by influencing the host’s gut microbiome (22, 23). At present, our understanding of the composition of the gut virome in birds, the functions of gut virome genes, and their metabolic interactions with the avian host is limited. Notably, the extensively studied pathogenic viruses currently constitute only a minor portion of the entire virosphere (24, 25). Moreover, certain viruses (phages), facilitated by auxiliary metabolic genes (AMGs), can directly impact host metabolism to enhance their environmental adaptability (24), particularly within migratory bird populations residing in extremely cold regions. Hence, determining the interaction between viruses and microbes can reveal how viruses influence the gut microbiota. Furthermore, the widespread use of antibiotics in human, veterinary, and agricultural practices leads to the continuously release of antibiotics and antibiotic-resistance genes (ARGs) into the environment (26), with phages significantly contributing to their spread (27), increasing pressure on public health. However, it is not confined to a regional or national issue; birds may play a role in its rapid global dissemination (28). Nevertheless, the ARGs originating from the gut of birds remain to be explored.

In this study, we employed viral metagenomics methods to focus on RNA viral sequences concealed within the cloaca of 2,990 wild and housed birds, aiming to determine their genetic relationships and assess the spill-over risk of these viruses to humans, livestock, and wildlife. Furthermore, we examined the novelty, functionality, and classification of the phages involved in this study.

## MATERIALS AND METHODS

### Sample collection and processing

From 2018 to 2019, cloacal samples of 2,990 birds (2,296 birds were wild, and 694 birds were housed in zoos, farms, or emergency centers) were collected using disposable absorbent cotton swabs (29). The samples were collected from 19 different regions of nine provinces in the Chinese mainland (Table S1). All samples were stored in sterile containers and shipped on dry ice. Before conducting viral metagenomics analysis, the tips of the collected swabs were immersed in 0.5 mL of Dulbecco’s phosphate-cushioned saline and vortexed for 5 min vigorously; then they were incubated at 4°C for 30 min. After centrifugation at 15,000 × *g* for 10 min, the supernatants were collected in 1.5 mL centrifuge tubes and stored at −80°C for later use (30). The birds that were caught using cannon nets were identified by experienced ornithologists. None of the birds showed any sign of disease. All samples were shipped to the Shanghai Veterinary Research Institute of the Chinese Academy of Agricultural Sciences where all experiments were carried out in accordance with the guidelines of the Biosafety Level 2 Laboratory at Shanghai Veterinary Research Institute (SVRI).

Each library consisted of fecal samples from the same bird species within the same regions. For each library, 100 µL of the supernatant was pipetted from 5 to 27 samples (3.7–20 µL per sample) and collected in a new 1.5 mL tube. These samples were centrifuged at 12,000 × *g* for 5 min at 4°C and filtered through a 0.45 µm filter to remove non-viral particles. RNase and DNase were used to treat the filtrates, followed by digestion of unprotected nucleic acids at 37°C for 60 min (31). Total nucleic acids were then extracted using the manufacturer’s protocol provided with the QIAamp MinElute Virus Spin Kit. These nucleic acid samples containing DNA and RNA viral sequences were used for reverse transcription reactions with the SuperScript III reverse transcriptase and 100 pmol of a random hexamer primer, followed by a single round of DNA synthesis using Klenow fragment polymerase. Libraries were constructed using the Nextera XT DNA Sample Preparation Kit and sequenced on the Illumina MiSeq or HiSeq platform with 250 base-paired ends with dual barcoding.

All steps in the experiment were performed, taking necessary measures to prevent sample cross-contamination and nucleic acid degradation during the process. Aerosol filter tips were used to reduce the probability of sample cross-contamination, and all other experimental materials, including microcentrifuge tubes and tips, which were in direct contact with nucleic acid samples, were free of DNase and RNase. The samples were dissolved in diethyl pyrocarbonate (DEPC)-treated water containing RNase inhibitors.

For blank controls, sterile ddH_2_O was prepared simultaneously and further processed under the same experimental conditions. Quality testing was performed using agarose gel electrophoresis and Agilent bioanalyzer 2100, and no DNA was detected in the control pool. While sequencing on the Illumina MiSeq or HiSeq platform, the control pool generated a very small number of reads. No viral sequences were found in the control pool when a BLASTx search was performed.

### Metagenome assembly and quality control

These 192 libraries corresponded to 82 different bird species (Table S1). Bowtie2 v2.4.5 (32) was used to align and remove potential host reads. Primers and low-quality sequences were trimmed using Trim Galore v0.6.5 (https://www.bioinformatics.babraham.ac.uk/projects/trim_galore), and the files were quality controlled with specific options as follows “--phred33 --length 35 --stringency 3 --fastqc --paired.” The quality control reports were then integrated using MultiQC (33) with default parameters. Duplicated reads were marked using PRINSEQ-lite v0.20.4 (-derep 1). All 192 data sets were assembled based on an in-house pipeline. Paired-end reads were assembled using MEGAHIT v1.2.9 (34) with default parameters. The results were then imported into Geneious Prime v2022.0.1 (https://www.geneious.com) for batch sequence renaming. To reduce false negatives during sequence assembly, further semi-automatic assembly was performed of the unmapped contigs and singlets that were <500 bp long, and contigs that were >1,500 bp long after reassembly were retained. Additionally, mixed assembly was performed using MEGAHIT combined with BWA v0.7.17 (35) to search for unused reads and low-abundance contigs. Individual contigs were used as the reference for mapping to the raw data using the Low Sensitivity/Fastest parameter in Geneious Prime.

### Searching for unknown RNA viruses in avian libraries

We identified RNA viral sequences in the avian libraries using the following steps: (i) a local viral sub-database containing the non-redundant protein (nr) database (download in May 2022) and IMG/VR v3 was constructed specifically for screening the assembled RNA viral contigs (36). The tentative RNA viral contigs were imported into Geneious Prime for manual assembly and inspection. Putative open-reading frames (ORFs) were predicted by Geneious Prime with default parameters (minimum size: 400; genetic code: standard; start codons: ATG) (37). We further checked them by comparing them to related viruses in the GenBank database. The annotations of these ORFs were based on comparisons to the Conserved Domain Database (CDD). (ii) The resulting sequences were selected based on those having less than 60% amino acid sequence identity to the best match, which was determined using a cut-off E-value of <10^–5^. (iii) The RNA virus data set was constructed using BWA v0.7.17 (38), and MMseqs2 was used to perform genome clustering (-k 0 -e 0.001 --min-seq-id 0.95 c 0.9 --cluster-mode 0) (39). (iv) The coverage of each unique RNA sequence was calculated using pileup, and the relative abundance of each sequence was obtained by a custom Bash shell script. (v) The TaxonKit (40) software was used for the taxonomic identification of RNA viruses.

### Phylogenetic analysis

To elucidate phylogenetic relationships, nucleotide and protein sequences of reference strains belonging to different groups of corresponding viruses were downloaded from the National Library of Medicine (NCBI) GenBank database, along with sequences of proposed species that have not yet been ratified. Related nucleotide and protein sequences were aligned using an alignment program implemented in the Qiagen CLC Genomics Workbench 10.0, and the resulting alignment was further optimized using MUSCLE in MEGA-X (41). Sites containing more than 50% gaps were temporarily removed from alignments. Maximum likelihood trees were then constructed using IQ-TREE (42). All phylogenetic trees were created using IQ-TREE with 1,000 bootstrap replicates (-bb 1000) and the ModelFinder function (-m MFP).

### Prediction of potential genome recombination events

In this study, potential recombination events were analyzed and filtered using the default algorithm of Recombination Detection Program version 4.39 (RDP4) software (43), which comprises of RDP, GENECONV, BootScan, MaxChi, Chimaera, SiScan, and 3Seq.

### Identification and clustering of phage genomes

Contigs were initially recognized in accordance with the viral sequence identification standard operating procedure (SOP) (https://doi.org/10.17504/protocols.io.bwm5pc86). In brief, contigs exceeding 5 kb were validated using VirSorter2 (44), and those passing the verification step were subjected to CheckV (45) to remove host sequences flanking prophages. RNA viral contigs were examined separately in the above section due to the higher false positive rates associated with currently available bioinformatics tools for identifying viral contigs under 5 kb (46, 47). The obtained genomes underwent screening using information from VirSorter2 and CheckV results, considering viral and host gene counts, VirSorter2 viral scores, and hallmark gene counts. The subset was subsequently clustered at 95% average nucleotide identity across 85% of the shortest contig per MIUViG standards (48), utilizing a custom script from the CheckV repository, resulting in phage populations. Phage populations were categorized into genus-level viral taxa through a gene-sharing network analysis using vContact2 (49), with NCBI RefSeq Viral (release 211) serving as the reference genomes. The clustered contig networks were displayed using Cytoscape v3.10.0 (50).

### Functional annotation of phages

The viral contigs’ ORFs were subjected to functional annotation by comparing them with the eggNOG v5.0 database using eggNOG-mapper v2 (51). Additionally, we aligned phage-associated protein sequences against the Comprehensive Antibiotic Resistance Database (CARD) using strict parameters, focusing solely on perfect and strict hits, to predict the profiles of ARGs (52). The prediction of viral host linkages and lifestyles was conducted using the PhaTYP and CHERRY suites within PhaBOX with default parameters (53).

## RESULTS

### Highly differentiated RNA viruses are present in the gut of birds

**Fig 1:**
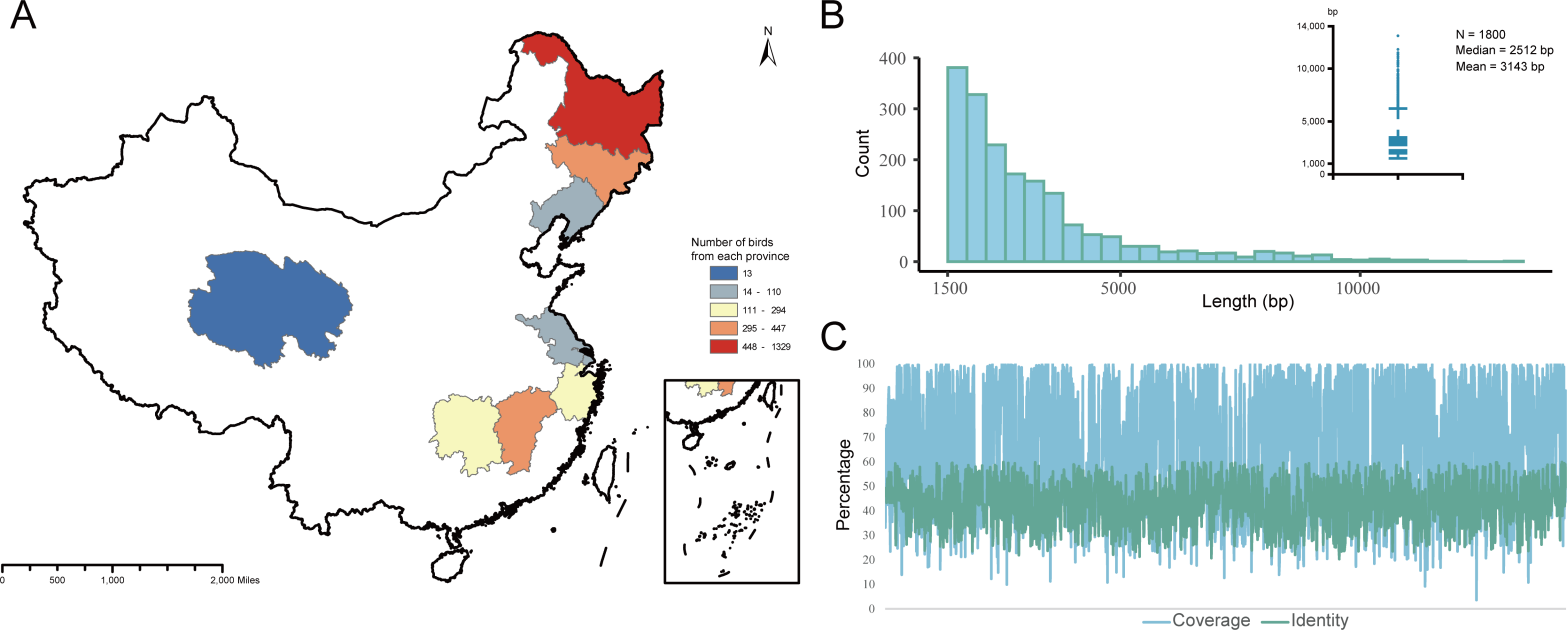
RNA virus data sets and associated collection sites included in this study. (**A**) A map showing the locations from which bird samples were collected. Different colors represent the number of samples corresponding to the legend. The source of the map is Geospatial Data Cloud (https://www.gscloud.cn), and the software used to create the map is ArcMap v10.5. All of these data are freely available to the public. (**B**) The distribution of viral contig length. The *x*-axis indicates the viral contig length; among 1,800 contigs, the median length was 2,512 bp. (**C**) The identity and coverage distribution of viral contigs with the best matches in the GenBank database.

**Fig 2:**
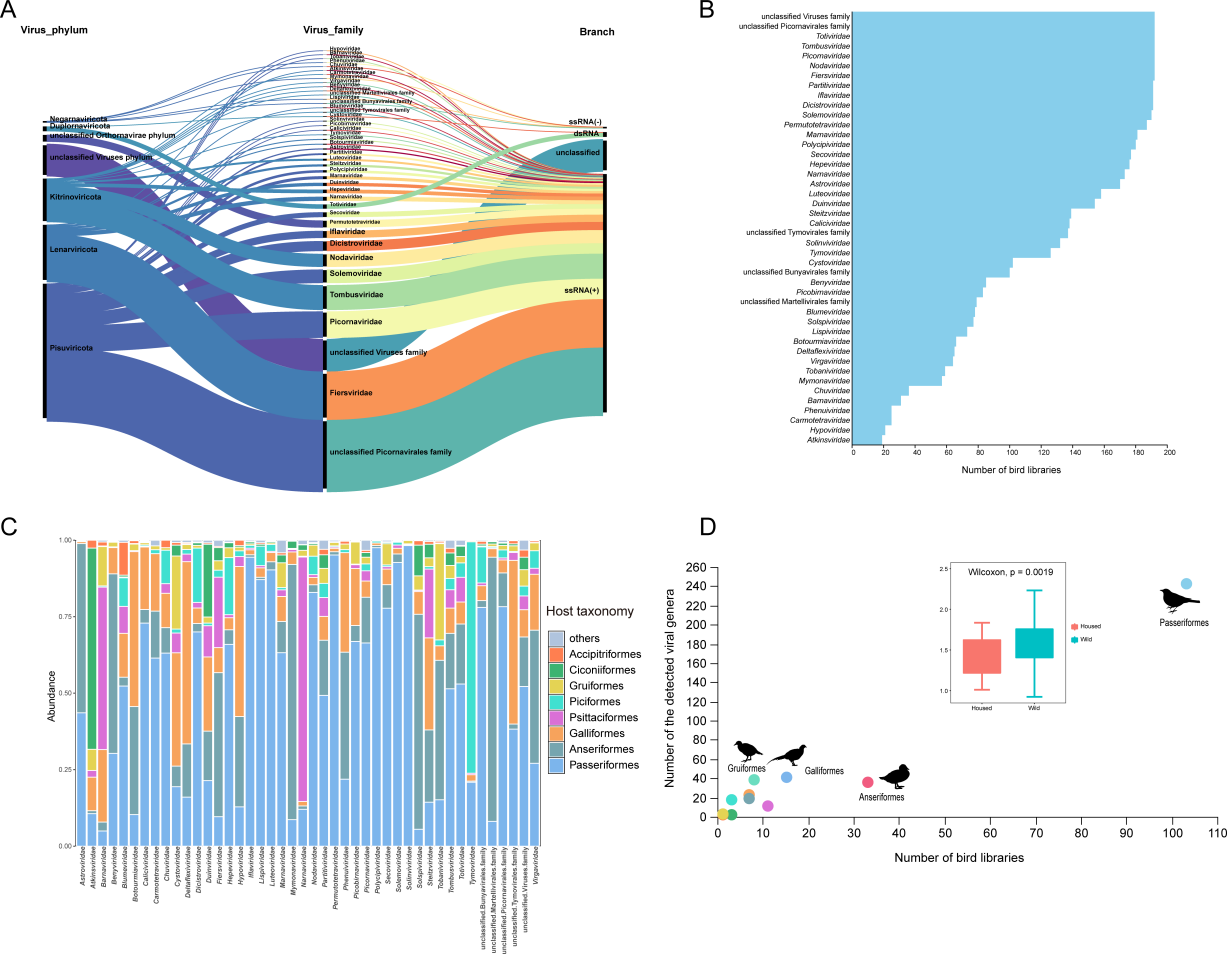
The diversity of RNA viruses identified in this study. (**A**) An alluvial plot depicting the distribution of taxa associated with 1,800 viral sequences. (**B**) The distribution of different viral families in avian libraries. (**C**) A histogram of the distribution of viral families in different orders of birds. (**D**) A scatter plot of the number of families of viruses present in different bird orders. The silhouettes of birds and other animals are sourced from https://www.phylopic.org/. All of these data are freely available to the public.

A large-scale viral metagenomics survey was performed by collecting cloacal swab samples of 2,990 birds from 19 different sampling sites in nine provinces of the Chinese mainland (Fig. 1A). We identified 1,800 sequences of previously undescribed RNA viruses associated with birds (Table S1), each sequence containing complete or near-complete RNA-dependent RNA polymerase (RdRp) coding DNA sequence and was validated by comparison with CDD. These RNA viral sequences were of different lengths, ranging from 1,500 bp to 13,101 bp. The median length of these viral contigs was 2,512 bp, and the coverage of these contigs with the best match ranged from 3.6% to 100% (Fig. 1B and C). These sequences were shorter than RNA viral reference genomes, which indicated that most viral contigs were detected as partial sequences of viral genomes. After taxonomic assignment, most of the sequences were positive-sense single-stranded RNA (+ssRNA) viruses. Specifically, these sequences were annotated into seven viral phyla, with the largest number of sequences belonging to the phylum *Pisuviricota* (882 sequences; 49.0%), followed by the phylum *Lenarviricota* (362 sequences; 20.1%) and the phylum *Kitrinoviricota* (278 sequences; 15.4%; Fig. 2A). At the family level, the number of sequences that were not annotated to a specific viral family was about 644 (35.8%), accounting for about one-third of the number of RNA viruses identified in this study, most of which were tentatively classified as members of the unclassified family Picornavirales. Seven viral families or groups (*Totiviridae*, *Tombusviridae*, *Picornaviridae*, *Nodaviridae*, *Fiersviridae*, the unclassified family Picornavirales, and the unclassified Viruses family) were detected in all 192 libraries (Fig. 2B; Table S2). Furthermore, all well-defined virus families identified in this study were found within the order *Passeriformes*. The abundance of these viruses in the orders *Anseriformes* and *Galliformes* was also significant, closely correlating with the number of birds collected (Fig. 2C).

We identified viruses belonging to the “unclassified” group by comparing the RdRp sequences of each virus with those of other viruses identified in this study. We defined a criterion: if a sequence shared less than 20% amino acid identity with all other viruses, it represented a novel genus of RNA viruses. This criterion is currently stricter than the classification standards for any RNA virus genus based on RdRp in the International Committee on Taxonomy of Viruses (ICTV; https://ictv.global/report). Based on this criterion, we identified 324 novel genera of RNA viruses. Among them, the unclassified Viruses family, unclassified Picornavirales family, unclassified Tymovirales family, unclassified Martellivirales family, and unclassified Bunyavirales family contained 66 (20.4%), 251 (77.5%), 3 (<1%), 2 (<1%), and 2 (<1%) viral genera, respectively (Table S3). We also estimated the potential viral genera present in each bird order, among which order *Passeriformes* contained the highest number of virus genera (242; see Table S4 for details). We also found that wild birds had higher RNA viral diversity than housed birds (Fig. 2D).

**Fig 3:**
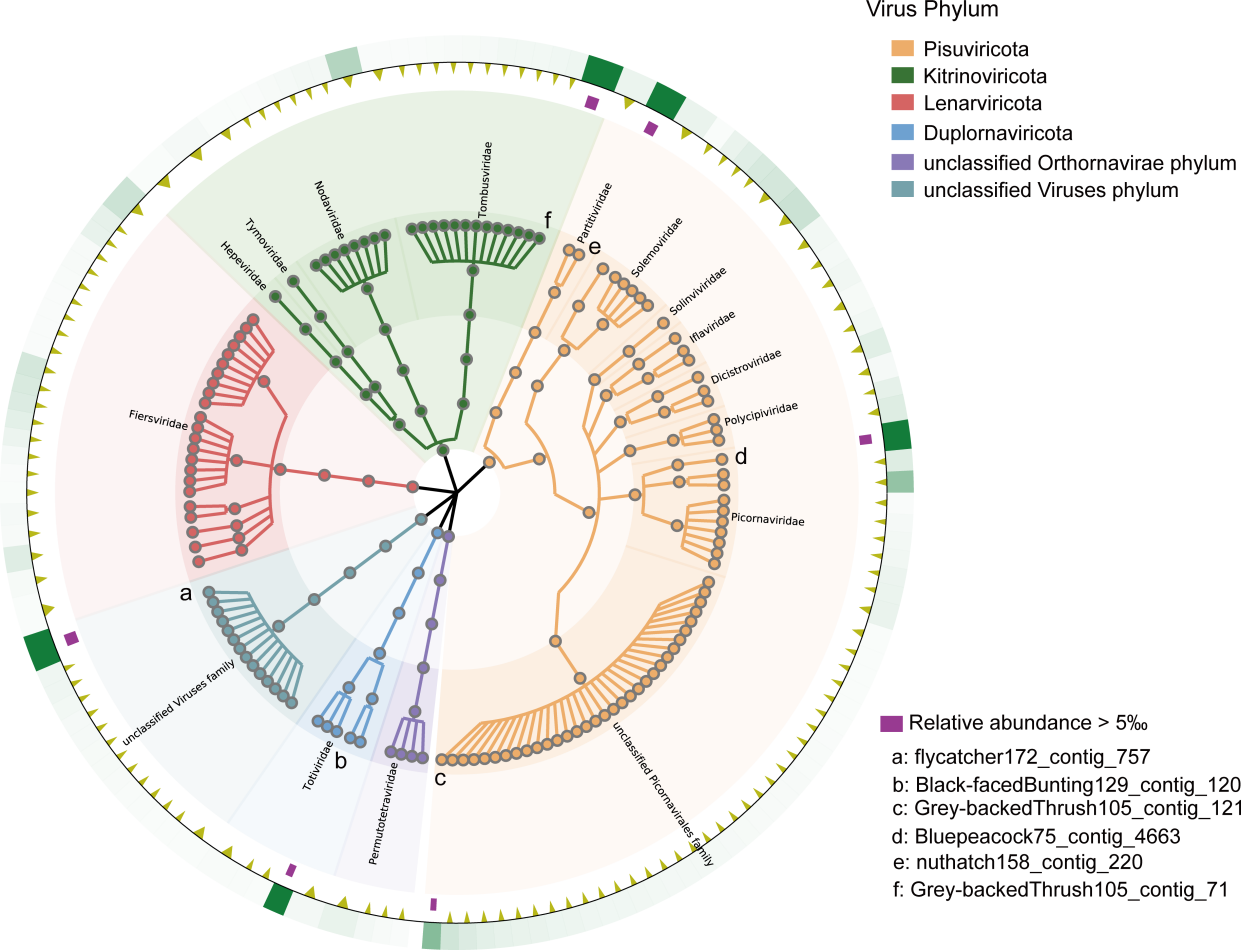
A GraPhlAn taxonomic tree related to the RNA viruses. The 130 most abundant RNA viruses in bird libraries were screened; different background colors represent different viral phyla. The outermost green squares represent the relative abundance heatmap for each viral species. The yellow triangles represent viruses with abundances below the 5‰ threshold of all viral quantities, while the purple squares represent those with abundances above 5‰.

To exclude the possibility of *de novo* assembly artifacts, we extracted the nucleotide sequences of the coding regions of these 1,800 sequences and mapped them to all collected libraries to compute coverage. We then screened the 130 most abundant RNA viruses across all libraries and constructed a GraPhlAn taxonomic tree (Fig. 3), where the abundances of six viruses exceeded 5‰ of the total detected RNA viruses (Table S5). Among them, flycatcher172_contig_757 and Grey-backedThrush105_contig_121 were identified as unclassified viruses. Black-facedBunting129_contig_120 was considered to belong to the genus *Totivirus*, and a BLASTx search showed that it had 58% amino acid sequence identity with the totivirus carried by green rice leafhopper (*Nephotettix cincticeps*) collected in rice fields. Nuthatch158_contig_220 and Grey-backedThrush105_contig_71 belonged to the families *Partitiviridae* and *Tombusviridae*, respectively, suggesting a close relationship between the birds and the plants or fungi they ate or came in contact with. Bluepeacock75_contig_4663, which was classified as a *Hepatovirus*, was detected as a part of a complete viral genome capable of infecting vertebrates. It shared 48% amino acid sequence identity with a virus (mute swan feces associated hepatovirus 3) identified in swans in the UK, suggesting that this virus and its relatives may have been spread worldwide through birds as a vector.

### Expansion of diversity in vertebrate-associated viruses

#### Astroviridae

**Fig 4:**
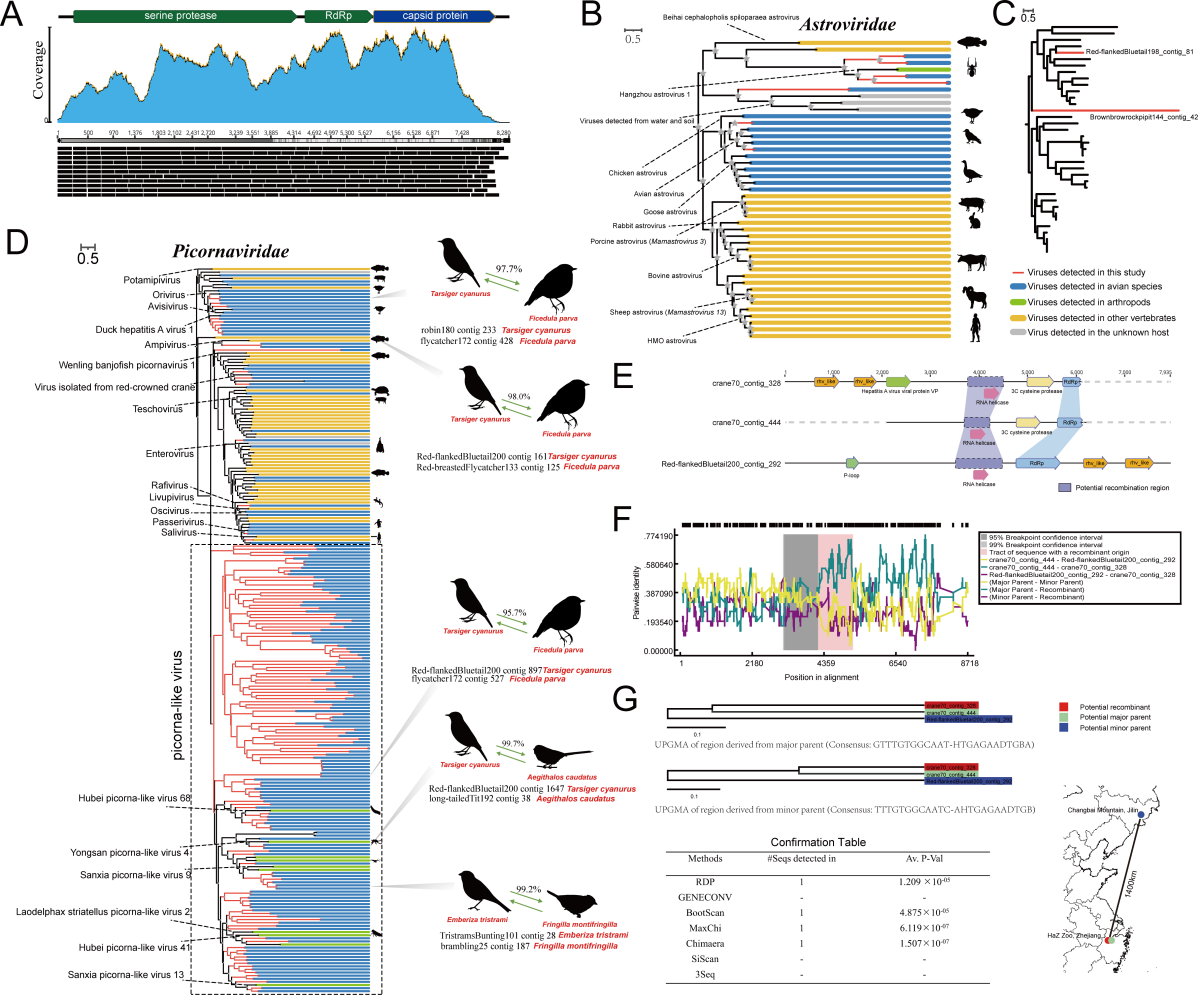
Novel astroviruses and picornaviruses identified in the cloaca of birds. (**A**) The complete genome organization of the novel astrovirus is illustrated using Brownbrowrockpipit144_contig_42 as an example. The top panel displays the three typical ORFs encoded by the novel astrovirus; the blue color below represents the coverage of each read of the virus against the reference genome of its library; the black box indicates the sequencing depth of each read. (**B**) The maximum likelihood tree based on astrovirus RdRp proteins. Branches in red indicate viruses newly identified in this study. (**C**) The maximum likelihood tree based on astrovirus capsid proteins. Red branches represent newly discovered viruses (containing both RdRp and Cap) in this study. (**D**) The maximum likelihood tree constructed based on the RdRp proteins of picornaviruses. The branches in red represent viruses identified in this study. (**E**) The genomic organization of RNA viruses involved in recombination analysis. (**F**) Results of potential recombination events analyzed using RDP4 software. (**G**) The upper panel shows the UPGMA analysis constructed between the putative recombinant and its major and minor parents in potential recombination events. The confirmation table below shows the support obtained by the algorithms for the identified recombination events. The simplified map on the right indicates the geographical location of the sequence libraries.

Viruses in the family *Astroviridae* comprise two genera identified in 2004, including the genus *Avastrovirus*, which infects birds, and the genus *Mamastrovirus*, which infects mammals (54). Most *Avastrovirus* species that infect domestic birds might cause some intestine-related diseases (55). We characterized seven astroviruses (including two complete genomes) from five different species of birds; the organization of a typical complete astrovirus genome is shown in Fig. 4A. The phylogenetic analysis of the protein sequences based on RdRp indicated that these seven astroviruses were divided into three clades. Two viruses from different bird species (Red-flankedBluetail198_contig_81 from *Tarsiger cyanurus* and Yellow-throatedBunting89_contig_41 from *Emberiza elegans*) clustered together with chicken/avian astrovirus, sharing less than 55% amino acid sequence identity with it (Fig. 4B). An astrovirus (BluePeacock205_contig_12951) from *Pavo cristatus* formed a distinct branch outside the astroviruses of unknown hosts detected from water and soil and shared <26% identity with the RdRp protein sequences of these astroviruses. The remaining four viruses are most closely related to Hangzhou astrovirus 1, which was isolated from *Tetragnatha nitens* collected from a rice field. According to the ICTV, the classification of *Avastrovirus* species is being redefined. Based on the genetic analysis of the complete capsid region at the amino acid level, avian astroviruses might be divided into two main genogroups, including genogroup I and genogroup II. The mean amino acid genetic distance (p-dist) between the genogroups is 0.704 ± 0.013. Based on the phylogenetic analysis and sequence analysis of the Cap protein, the genetic distance between the two complete viruses identified in this study and their closest strains was 0.685–0.922, indicating that novel genogroups might exist (Fig. 4C; Table S6). The topological structures of the phylogenetic trees constructed based on the Cap protein and the RdRp protein were very similar, indicating that these astroviruses did not undergo recombination.

#### Picornaviridae

According to the ICTV, there are at least 68 genera of viruses in the family *Picornaviridae*, and these viruses can cause widespread infection in many species, including humans. Picornaviruses were among the most prevalent viruses found in the bird species examined. We obtained 158 novel picornaviruses from 74 libraries, and their amino acid sequence identity with the best matches ranged from 20.3% to 59.7%. The results of the phylogenetic analysis based on the RdRp protein sequences showed that some of these picornaviruses were closely related to the defined genera *Potamipivirus*, *Oscivirus*, *Enterovirus*, *Hepatovirus*, *Livupivirus*, *Orivirus*, and *Ampivirus* (Fig. 4D). Most of the remaining viruses, which were considerably different from the known picornavirus RdRp proteins, were temporarily classified as picorna-like viruses, potentially forming several novel genera or families, with some of the viruses being associated with arthropods such as mosquitoes, shrimps, and lice. Additionally, sequence analysis suggested the possibility of cross-species transmission of these picornaviruses. Interestingly, a clear recombination signal was identified in the RNA helicase region of crane70_contig_328, which was detected in the feces of *Grus japonensis* housed in the Hangzhou Zoo in Zhejiang, China. The major parent was identified as crane70_contig_444, while the minor parent was identified as Red-flankedBluetail200_contig_292, which was detected in wild *T. cyanurus* in Changbai Mountain, Jilin, China (Fig. 4E and F). This result was supported by four algorithms (RDP, BootScan, MaxChi, Chimaera; Fig. 4G).

#### Phenuiviridae

**Fig 5:**
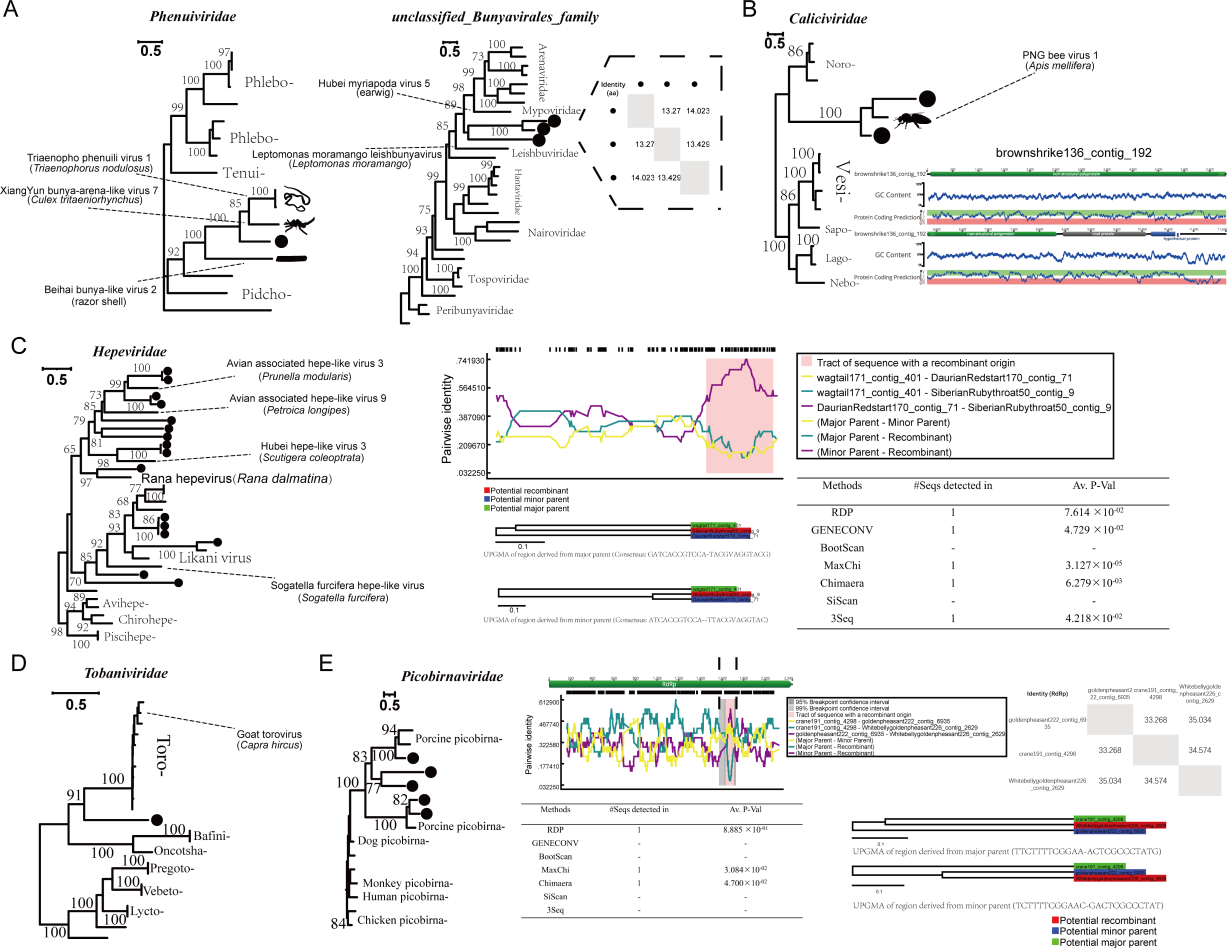
RNA viruses associated with vertebrates. (**A**) The maximum likelihood trees were constructed based on the RdRp proteins of phenuiviruses and unclassified_Bunyavirales_family; the dashed box represents the amino acid identity in the RdRp among the three viruses clustered together. The black dots represent the viruses found in this study. (**B**) Phylogenetic analysis based on the RdRp protein of the family *Caliciviridae*, with the complete genome organization of one of the obtained viruses shown on the right. (**C**) The maximum likelihood tree constructed based on the RdRp proteins of hepeviruses; the potential recombination events are shown on the right. (**D**) The maximum likelihood tree constructed based on the RdRp proteins of tobaniviruses. (**E**) The maximum likelihood tree constructed based on the RdRp proteins of picobirnaviruses; the potential recombination events are shown on the right.

Members of the family *Phenuiviridae* might cause zoonotic diseases, such as self-limiting fever, retinitis, and severe hemorrhagic fever (56, 57). A novel virus (blackswan219_contig_10769) from *Cygnus atratus* belonging to the order *Anseriformes* was identified. It contained the complete RdRp protein related to the family *Phenuiviridae* (4,593 nt; 1531 aa; Fig. 5A) and shared about 38.3% amino acid sequence identity with the best match (Triaenopho phenuili virus 1 isolated from *Triaenophorus nodulosus*). Based on the phylogenetic relationship, this virus was not related to any known genus. Additionally, three sequences belonging to the unclassified Bunyavirales family were identified, which formed a separate clade between the families *Leishbuviridae* and *Mypoviridae*.

#### Caliciviridae

Caliciviruses can cause various diseases, including gastroenteritis, respiratory infections, and reproductive failure in humans and animals. Many unclassified novel caliciviruses were recently detected in various animals, including wild birds (58), geese (59), and fish (60). We identified two strains belonging to the family *Caliciviridae*, one of which is a complete genome (brownshrike136_contig_192) with a length of 11,628 nt. It encodes two major proteins and one protein with an unknown function. They did not cluster with any known genera, and the closest virus to them in the public database was PNG bee virus 1, isolated from the Western honeybee (*Apis mellifera*; Fig. 5B).

#### Hepeviridae

The family *Hepeviridae* can infect mammals, birds, and fish (61). The viruses from this group originated due to recombination between the capsid region of the chicken astrovirus and the ORF1 region of *Alphatetraviridae* (62). We identified 16 viruses belonging to the family *Hepeviridae*, and except for wagtail171_contig_401, which was closely related to the Rana hepevirus, the remaining 15 viruses did not cluster significantly with any known genus (Fig. 5C). Furthermore, we have identified recombination signals in the RNA helicase region of SiberianRubythroat50_contig_9. Interestingly, its major parent was from the RdRp region of wagtail171_contig_401, while its minor parent was from the RNA helicase region of DaurianRedstart170_contig_71.

#### Tobaniviridae

*Tobaniviridae* can infect vertebrates, such as pigs, cows, and even humans (63). A novel virus containing the complete RdRp protein was detected from *G. japonensis*, and sequence analysis suggested that the virus belonged to the family *Tobaniviridae*. The results of the phylogenetic analysis indicated that the virus was closely related to the genus *Torovirus*, which was earlier classified as a coronavirus. *Torovirus* is rarely detected in bird hosts. In this study, the virus shared 39% amino acid sequence identity with the best match (goat torovirus; YP_009380535), suggesting that birds could potentially serve as vectors for the transmission of this virus (Fig. 5D).

#### Picobirnaviridae

*Picobirnaviridae* may infect mammals and invertebrates and cause associated symptoms, including gastroenteritis in animals and humans (64). Five viruses related to *Picobirnaviridae* were identified from four avian hosts (*Pavo cristatus*, *Grus monacha*, *Chrysolophus pictus*, and *Chrysolophus amherstiae*). The results of the phylogenetic analysis of the RdRp protein showed that they clustered with picobirnaviruses identified from pigs, indicating a close relationship between birds and domestic animals (Fig. 5E). Similarly, we have also detected evidence of recombination in the coding region of Whitebellygoldenpheasant226_contig_2629, with a minimum length of 60 bp.

### Diversity of phages in the gut of wild birds

**Fig 6:**
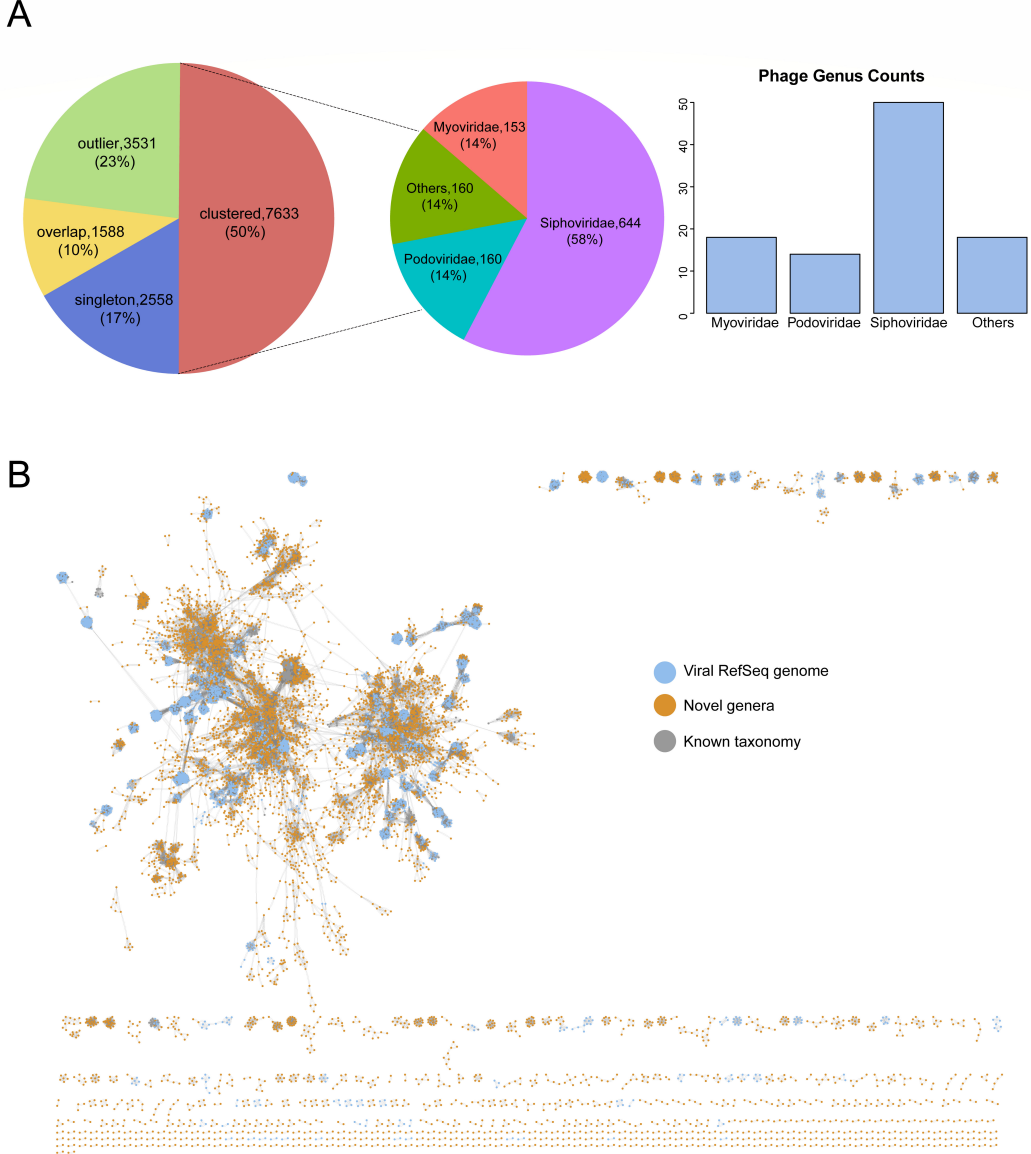
Recovered phage contigs in the gut of wild birds. (**A**) The left pie chart showed the distribution of phage contigs according to their clustering result based on their shared proteins using vConTACT2. The right pie chart displayed the distribution of phage populations assigned to known viral families, and the bar chart revealed the number of phage populations that could be classified into known genera. (**B**) Viral clusters from gene-content-based network analysis. The node coloring is as indicated by the legend on the right side of the figure.

We have identified 21,131 phage contigs, each exceeding 5 kb, and subsequently clustered them into 15,310 phage populations. Among these, 5,132 (33.5%) populations exceed 10 kb. Upon verification using CheckV, it was determined that 452 (3.0%) populations are complete, 842 (5.5%) are of high quality, and 1,076 (7.0%) are of medium quality. Roughly 40% of phage populations were identified as singletons or outliers. Ten percent of phage populations were represented as overlaps, indicating they were ascribed to more than one cluster, obscuring their cluster affiliation (Fig. 6A). We then conducted protein clustering of our exclusive 15,310 populations using original vContact2 database, unveiling 2,841 viral clusters, encompassing 50% of our phage populations (Table S7). The network comprises 2,740 modules and 956 edges, which were streamlined to display solely protein clusters containing at least one phage population from our investigation. We classified 100 phage populations (0.7%) as belonging to established genera and 1,117 phage populations (7.4%) to recognized families. The majority of assignable phage populations (85.7%) were attributed to *Siphoviridae*, *Myoviridae*, or *Podoviridae* within the *Caudovirales* order. The remaining phage populations were unable to be allocated to any known genera or families, indicating the presence of novel lineages (Fig. 6B).

### Predicted hosts and lifestyles for phage genomes

**Fig 7:**
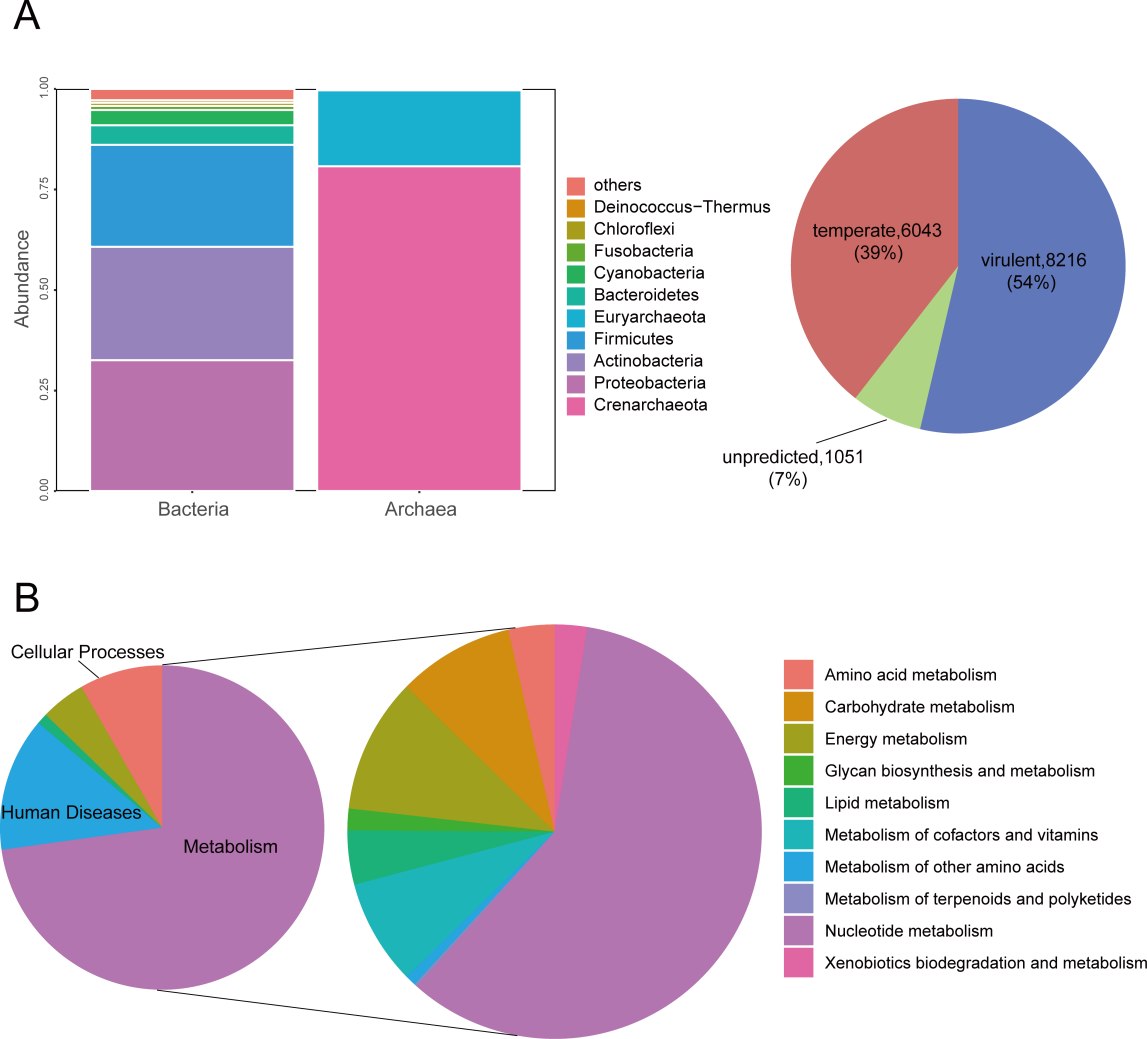
Phage host prediction and functional gene analysis. (**A**) The bar chart revealed the host distribution of different phage populations, while the pie chart displayed the predicted lifestyles of these phage populations. (**B**) The left pie chart displayed the distribution of phage populations assigned to different Kyoto Encyclopedia of Genes and Genomes (KEGG) pathways, while the right pie chart showed the distribution of phage populations across various metabolic pathways.

Among the 15,310 phage populations, hosts could be reliably predicted for 12,294 (80.3%) genomes. The vast majority of predicted hosts belonged to bacteria (*n* = 10,574; 86.0%), with a small portion belonging to archaea (Table S8). These hosts are distributed among 30 different bacterial phyla, with the most abundant being *Proteobacteria* (3,439 populations; 32.5%), followed by *Actinobacteria* (2,980 populations; 28.2%) and *Firmicutes* (2,677 populations; 25.3%). In general, these three phyla dominate across all species. *Crenarchaeota* and *Euryarchaeota* phyla constitute the largest proportions within archaea (Fig. 7A). Additionally, 54% of these populations are predicted to comprise virulent phages, while 39% are predicted to be temperate phages. It is noteworthy that virulent phages in the gut of wild birds tend to possess larger genome sizes and higher guanine-cytosine (GC) content, as determined by the Mann-Whitney Test (*P* = 0.000). Interestingly, in a systematic analysis concerning marine lysogens and proviruses, marine lysogens (those infected by temperate phages) often exhibited significantly larger genome sizes and higher GC content compared to nonlysogens (65).

### Identification of putative AMGs and ARGs

In addition to its impact on the microbial community, viruses can also regulate host metabolism through AMGs. From the avian gut phage data set, 6,625 unique AMGs were detected, among which there were 622 unique Gene IDs (Table S9). These genes encompass multiple functional categories. Approximately 70% of the genes were related to metabolic pathways, encoding enzymes involved in a wide range of metabolic processes, including nucleotide, lipid, vitamin, carbohydrate, and amino acid metabolism (Fig. 7B). Among these AMGs, we found ssb to be the most common. It is capable of participating in various aspects of single-stranded DNA metabolism, including replication, repair, and recombination. Besides, the AMG-encoded DNMT1, a DNA (cytosine-5)-methyltransferase that shields viruses from the antiviral restriction-modification systems of their hosts (66), was identified in nearly 200 phage populations. This type of AMG potentially acts as a phage defense mechanism. Alongside directly boosting host nucleotide metabolism to facilitate virus replication, certain viruses utilize AMGs to promote host nutrient acquisition, thereby indirectly improving virus survival rates (47). Furthermore, we only identified a single type of ARG from 14 phage populations, the vanY gene, which falls within the glycopeptide antibiotic class. The vanY gene functions as a D,D-carboxypeptidase, responsible for removing the terminal D-Ala from peptidoglycan to facilitate the incorporation of D-lactate (Table S9). Compared to recent research, the number and diversity of ARGs carried by phages in the gut of birds are significantly lower than those carried by bacteria in the gut of birds (28). Although ARGs are rarely encoded in phages (67), there have also been studies exploring a certain number of ARGs carried by avian gut phages (68).

## DISCUSSION

The diversity of viruses is extremely high, and almost all biological cells might be infected by viruses. The diversity of hosts, the instability of the viral structures (especially RNA viruses), and the propensity to exchange genetic material with other host viruses contribute to the unparalleled diversity of viral genomes (69). Recent advancements in metagenomics have provided a lot of genetic information on viruses; however, many things are still unknown. There are at least 40,000 different viral species in mammals, as determined by some studies, which far exceeds the viral species currently identified by the ICTV (70). Therefore, continuous and extensive research on viral diversity is required for dealing with future epidemics. The sampling of viruses from a larger diversity of avian hosts should provide better evolutionary insights.

Birds, especially migratory birds, are important repositories of viruses. Like other animals, they often carry multiple pathogens such as viruses, mycoplasma, chlamydia, bacteria, and various parasites. During migration, birds pollute water sources and encounter poultry raised by humans and other wild animals, thus spreading pathogens and diseases, such as the *West Nile virus* (71) and avian influenza A virus (HPAI H5N1 Asian lineage) (72), to other animals and even humans. The largest number of birds found in this survey belonged to the order *Passeriformes* (about 1,551 individuals). *Passeriformes* is the most complex order of birds and is found in every continent except Antarctica. They mainly inhabit forests, farmland, parks, and residential areas. Hence, the viruses they carry might threaten mammals, including humans. Here, we identified a novel virus in the family *Phenuiviridae*, carried by the black swan (*C. atratus*). *Phenuiviridae* can infect humans via tick bites or by direct contact with animals, such as dogs, sheep, or chickens, bitten by ticks. Although there is currently no evidence of replication of this virus in birds, the risk of zoonotic diseases in areas where the infected birds migrate to should be assessed. In this study, we identified many vertebrate-associated RNA viruses, some of which are rarely found in birds. Based on this, we hypothesized that one or more species in the region act as vectors and spread the viruses to birds and other animals, thus helping these viruses expand their host range.

Before the field of metagenomics was established, information was limited on the viral diversity of samples that lacked health or economic interest to humans or livestock, such as invertebrates, plants, or other environmental samples. However, we now know that natural viral communities present in these species can be enormous (73). Birds greatly facilitate the interregional transmission of these viruses. In this study, we detected hundreds of RNA sequences related to plants, fungi, or insects. Viruses from families such as *Tombusviridae* (148), *Solemoviridae* (81), *Nodaviridae* (78), and *Dicistroviridae* (60) were relatively more abundant. These viruses are believed to be associated with plants or invertebrates, potentially reflecting the habitat and dietary preferences of birds. These viruses occupied topological positions on the evolutionary trees and have led to the formation of several new putative families, genera, or species, thereby promoting the phylogenetic continuity of the related viruses (Fig. S1). Some viruses might be carried by birds and cause new crop diseases in the areas where they migrate. Therefore, regular large-scale avian virus surveillance is needed in areas where crops are the main source of income. Additionally, we have identified hundreds of RNA sequences that cannot be assigned to any known viral catalog, indicating significant variation between these sequences and known viruses. Further investigation is required to determine whether these sequences can infect birds and cause diseases by replicating in the host, although no signs of disease were observed in any of the bird samples examined in this study. Although many studies have investigated the diversity of RNA viruses in birds, more research is still required in this field. Recent surveys have revealed an extremely high diversity of RNA viruses in urban sewage (74), marine plankton (75), and large populations of invertebrates (76). Information on the composition of the entire RNA virosphere is extremely limited. Although numerous samples have been examined, most of these sequences are not characteristic for researchers. It also challenges the established classification methods of viruses, such as how to define the fuzzy boundaries between virus families, which are sometimes very “broad” and might require a large number of sequences to fill in. This high diversity needs to be elucidated, and RNA viruses need to be studied in a larger ecological context.

Recently, a series of studies have identified a diverse array of viral particles in several ecosystems such as marine environments (77), the human gut (78), and soil (79). These investigations have led to the discovery of numerous novel viruses and AMGs, offering new insights into the ecological impacts of viruses. In this study, only about 7% of phage populations could be classified into known families or genera, with the majority being *Caudoviricetes*. However, in 2022, the ICTV abolished morphology-based taxa and change to binomial species names, resulting in the removal of taxa such as the order *Caudovirales* and the families *Myoviridae*, *Siphopviridae*, and *Podoviridae*. Therefore, morphology-based phage classification is not reliable. We associated these viruses with their hosts, some of which exhibited broad host characteristics. Additionally, we identified a large number of AMGs related to virus replication and host resistance. These characteristics may facilitate the viruses’ survival in such a specialized, nutrient-poor environment. Yet, most of the viral diversity, host associations, and functional involvement in the gut microbiota environment have not been thoroughly investigated. Hence, conducting a more extensive examination of viral diversity and interactions with microbial hosts, including aspects like host adaptation and lifestyle, could reveal the functional roles of microbes within this system. However, this study has some limitations: (i) non-viral particles were filtered out during sample processing, potentially leading to the oversight of viruses found within cells. (ii) Contigs shorter than 1,500 bp were discarded; thus, short viral RNA molecules might have been overlooked. (iii) The establishment of a threshold of 60% amino acid sequence identity may also result in the omission of some new viruses. Overall, this study not only aids in enhancing our understanding of gut viral communities of wild birds but also offers valuable resources for future virus research.

## Data Availability

The libraries and RNA viral sequences involved in this study have been deposited into the SRA and GenBank databases; please refer to Table S1 for details. Additionally, the phage genome data have been deposited in the Zenodo repository (https://doi.org/10.5281/zenodo.10644269) without any access restrictions.

## SUPPLEMENTAL MATERIAL

Figure S1 spectrum.00802-24-s0001.pdf The maximum likelihood trees of different RNA viruses.

Table S1 spectrum.00802-24-s0002.xlsx Details of the 192 bird libraries and the 1,800 RNA viruses.

Table S2 spectrum.00802-24-s0003.xlsx Family-level distribution statistics of RNA viruses in bird libraries.

Table S3 spectrum.00802-24-s0004.xlsx Representative sequence for putatively expanding genera of RNA viruses.

Table S4 spectrum.00802-24-s0005.xlsx RNA viruses information at the genus level detected in different bird orders.

Table S5 spectrum.00802-24-s0006.xlsx Relative abundance of RNA viruses in different bird libraries.

Table S6 spectrum.00802-24-s0007.xlsx The p-dist matrix containing two newly identified astroviruses.

Table S7 spectrum.00802-24-s0008.xlsx The detailed information of 2,841 viral clusters.

Table S8 spectrum.00802-24-s0009.xlsx The prediction results of host and lifestyle for phage genomes.

Table S9 spectrum.00802-24-s0010.xlsx The detailed information of 6,625 identified AMGs and 14 ARGs.

ASM does not own the copyrights to Supplemental Material that may be linked to, or accessed through, an article. The authors have granted ASM a non-exclusive, world-wide license to publish the Supplemental Material files. Please contact the corresponding author directly for reuse.

## References

[1] Camarillo-Guerrero LF, Almeida A, Rangel-Pineros G, Finn RD, Lawley TD. 2021. Massive expansion of human gut bacteriophage diversity. Cell 184:1098–1109. doi:10.1016/j.cell.2021.01.029 33606979 PMC7895897

[2] Zhang YZ, Shi M, Holmes EC. 2018. Using metagenomics to characterize an expanding virosphere. Cell 172:1168–1172. doi:10.1016/j.cell.2018.02.043 29522738

[3] Chen Y-M, Sadiq S, Tian J-H, Chen X, Lin X-D, Shen J-J, Chen H, Hao Z-Y, Wille M, Zhou Z-C, Wu J, Li F, Wang H-W, Yang W-D, Xu Q-Y, Wang W, Gao W-H, Holmes EC, Zhang Y-Z. 2022. RNA viromes from terrestrial sites across China expand environmental viral diversity. Nat Microbiol 7:1312–1323. doi:10.1038/s41564-022-01180-2 35902778

[4] Reed KD, Meece JK, Henkel JS, Shukla SK. 2003. Birds, migration and emerging zoonoses: west nile virus, lyme disease, influenza A and enteropathogens. Clin Med Res 1:5–12. doi:10.3121/cmr.1.1.5 15931279 PMC1069015

[5] Canuti M, Kroyer ANK, Ojkic D, Whitney HG, Robertson GJ, Lang AS. 2019. Discovery and characterization of novel RNA viruses in aquatic North American wild birds. Viruses 11:768. doi:10.3390/v11090768 31438486 PMC6784231

[6] Sekercioğlu CH, Daily GC, Ehrlich PR. 2004. Ecosystem consequences of bird declines. Proc Natl Acad Sci U S A 101:18042–18047. doi:10.1073/pnas.0408049101 15601765 PMC539768

[7] Viana DS, Santamaría L, Figuerola J. 2016. Migratory birds as global dispersal vectors. Trends Ecol Evol 31:763–775. doi:10.1016/j.tree.2016.07.005 27507683

[8] Yin R, Zhang P, Liu X, Chen Y, Tao Z, Ai L, Li J, Yang Y, Li M, Xue C, Qian J, Wang X, Chen J, Li Y, Xiong Y, Zhang J, Stoeger T, Bi Y, Chen J, Ding Z. 2017. Dispersal and transmission of avian paramyxovirus serotype 4 among wild birds and domestic poultry. Front Cell Infect Microbiol 7:212. doi:10.3389/fcimb.2017.00212 28603697 PMC5445105

[9] Ramey AM, Reeves AB, Sonsthagen SA, TeSlaa JL, Nashold S, Donnelly T, Casler B, Hall JS. 2015. Dispersal of H9N2 influenza A viruses between East Asia and North America by wild birds. Virology 482:79–83. doi:10.1016/j.virol.2015.03.028 25827532

[10] Ferguson NM, Fraser C, Donnelly CA, Ghani AC, Anderson RM. 2004. Public health. Public health risk from the avian H5N1 influenza epidemic. Science 304:968–969. doi:10.1126/science.1096898 15143265

[11] Keawcharoen J, van Riel D, van Amerongen G, Bestebroer T, Beyer WE, van Lavieren R, Osterhaus A, Fouchier RAM, Kuiken T. 2008. Wild ducks as long-distance vectors of highly pathogenic avian influenza virus (H5N1). Emerg Infect Dis 14:600–607. doi:10.3201/eid1404.071016 18394278 PMC2570914

[12] Li KS, Guan Y, Wang J, Smith GJD, Xu KM, Duan L, Rahardjo AP, Puthavathana P, Buranathai C, Nguyen TD, Estoepangestie ATS, Chaisingh A, Auewarakul P, Long HT, Hanh NTH, Webby RJ, Poon LLM, Chen H, Shortridge KF, Yuen KY, Webster RG, Peiris JSM. 2004. Genesis of a highly pathogenic and potentially pandemic H5N1 influenza virus in eastern Asia. Nature 430:209–213. doi:10.1038/nature02746 15241415

[13] Hamer SA, Lehrer E, Magle SB. 2012. Wild birds as sentinels for multiple zoonotic pathogens along an urban to rural gradient in greater Chicago, Illinois. Zoonoses Public Health 59:355–364. doi:10.1111/j.1863-2378.2012.01462.x 22353581

[14] Hubálek Z. 2004. An annotated checklist of pathogenic microorganisms associated with migratory birds. J Wildl Dis 40:639–659. doi:10.7589/0090-3558-40.4.639 15650082

[15] Dolan PT, Whitfield ZJ, Andino R. 2018. Mechanisms and concepts in RNA virus population dynamics and evolution. Annu Rev Virol 5:69–92. doi:10.1146/annurev-virology-101416-041718 30048219 PMC13283306

[16] Carroll D, Daszak P, Wolfe ND, Gao GF, Morel CM, Morzaria S, Pablos-Méndez A, Tomori O, Mazet JAK. 2018. The global virome project. Science 359:872–874. doi:10.1126/science.aap7463 29472471

[17] An D, Zhang J, Yang J, Tang Y, Diao Y. 2020. Novel goose-origin astrovirus infection in geese: the effect of age at infection. Poult Sci 99:4323–4333. doi:10.1016/j.psj.2020.05.041 32867976 PMC7598121

[18] Ao Y, Zhou Y, Li D, Duan Z. 2020. A novel picornavirus identified in wild Macaca mulatta in China. Arch Virol 165:495–504. doi:10.1007/s00705-019-04442-3 31845155

[19] Bodewes R. 2018. Novel viruses in birds: flying through the roof or is a cage needed? Vet J 233:55–62. doi:10.1016/j.tvjl.2017.12.023 29486880

[20] Chan J-W, To K-W, Chen H, Yuen K-Y. 2015. Cross-species transmission and emergence of novel viruses from birds. Curr Opin Virol 10:63–69. doi:10.1016/j.coviro.2015.01.006 25644327 PMC7102742

[21] Wille M, Eden JS, Shi M, Klaassen M, Hurt AC, Holmes EC. 2018. Virus-virus interactions and host ecology are associated with RNA virome structure in wild birds. Mol Ecol 27:5263–5278. doi:10.1111/mec.14918 30375075 PMC6312746

[22] Ritz NL, Draper LA, Bastiaanssen TFS, Turkington CJR, Peterson VL, van de Wouw M, Vlckova K, Fülling C, Guzzetta KE, Burokas A, Harris H, Dalmasso M, Crispie F, Cotter PD, Shkoporov AN, Moloney GM, Dinan TG, Hill C, Cryan JF. 2024. The gut virome is associated with stress-induced changes in behaviour and immune responses in mice. Nat Microbiol 9:359–376. doi:10.1038/s41564-023-01564-y 38316929 PMC10847049

[23] Ji Y, Xi H, Chen C, Sun C, Feng X, Lei L, Han W, Gu J. 2024. The pig intestinal phageome is an important reservoir and transfer vector for virulence genes. Sci Total Environ 916:170076. doi:10.1016/j.scitotenv.2024.170076 38220020

[24] Ettinger CL, Saunders M, Selbmann L, Delgado-Baquerizo M, Donati C, Albanese D, Roux S, Tringe S, Pennacchio C, Del Rio TG, Stajich JE, Coleine C. 2023. Highly diverse and unknown viruses may enhance Antarctic endoliths' adaptability. Microbiome 11:103. doi:10.1186/s40168-023-01554-6 37158954 PMC10165816

[25] Chen Y-M, Hu S-J, Lin X-D, Tian J-H, Lv J-X, Wang M-R, Luo X-Q, Pei Y-Y, Hu R-X, Song Z-G, Holmes EC, Zhang Y-Z. 2023. Host traits shape virome composition and virus transmission in wild small mammals. Cell 186:4662–4675. doi:10.1016/j.cell.2023.08.029 37734372

[26] Pazda M, Kumirska J, Stepnowski P, Mulkiewicz E. 2019. Antibiotic resistance genes identified in wastewater treatment plant systems - a review. Sci Total Environ 697:134023. doi:10.1016/j.scitotenv.2019.134023 31479900

[27] Pires J, Santos R, Monteiro S. 2023. Antibiotic resistance genes in bacteriophages from wastewater treatment plant and hospital wastewaters. Sci Total Environ 892:164708. doi:10.1016/j.scitotenv.2023.164708 37315610

[28] Cao J, Hu Y, Liu F, Wang Y, Bi Y, Lv N, Li J, Zhu B, Gao GF. 2020. Metagenomic analysis reveals the microbiome and resistome in migratory birds. Microbiome 8:26. doi:10.1186/s40168-019-0781-8 32122398 PMC7053137

[29] Shan T, Yang S, Wang H, Wang H, Zhang J, Gong G, Xiao Y, Yang J, Wang X, Lu J, et al.. 2022. Virome in the cloaca of wild and breeding birds revealed a diversity of significant viruses. Microbiome 10:60. doi:10.1186/s40168-022-01246-7 35413940 PMC9001828

[30] Wang H, Ling Y, Shan T, Yang S, Xu H, Deng X, Delwart E, Zhang W. 2019. Gut virome of mammals and birds reveals high genetic diversity of the family Microviridae. Virus Evol 5:vez013. doi:10.1093/ve/vez013 31191981 PMC6555873

[31] Zhang W, Li L, Deng X, Kapusinszky B, Pesavento PA, Delwart E. 2014. Faecal virome of cats in an animal shelter. J Gen Virol 95:2553–2564. doi:10.1099/vir.0.069674-0 25078300 PMC4202271

[32] Langmead B, Salzberg SL. 2012. Fast gapped-read alignment with Bowtie 2. Nat Methods 9:357–359. doi:10.1038/nmeth.1923 22388286 PMC3322381

[33] Ewels P, Magnusson M, Lundin S, Käller M. 2016. MultiQC: summarize analysis results for multiple tools and samples in a single report. Bioinformatics 32:3047–3048. doi:10.1093/bioinformatics/btw354 27312411 PMC5039924

[34] Li D, Luo R, Liu C-M, Leung C-M, Ting H-F, Sadakane K, Yamashita H, Lam T-W. 2016. MEGAHIT v1.0: a fast and scalable metagenome assembler driven by advanced methodologies and community practices. Methods 102:3–11. doi:10.1016/j.ymeth.2016.02.020 27012178

[35] Li H, Durbin R. 2010. Fast and accurate long-read alignment with Burrows-Wheeler transform. Bioinformatics 26:589–595. doi:10.1093/bioinformatics/btp698 20080505 PMC2828108

[36] Roux S, Páez-Espino D, Chen I-M, Palaniappan K, Ratner A, Chu K, Reddy TBK, Nayfach S, Schulz F, Call L, Neches RY, Woyke T, Ivanova NN, Eloe-Fadrosh EA, Kyrpides NC. 2021. IMG/VR v3: an integrated ecological and evolutionary framework for interrogating genomes of uncultivated viruses. Nucleic Acids Res 49:D764–D775. doi:10.1093/nar/gkaa946 33137183 PMC7778971

[37] Kearse M, Moir R, Wilson A, Stones-Havas S, Cheung M, Sturrock S, Buxton S, Cooper A, Markowitz S, Duran C, Thierer T, Ashton B, Meintjes P, Drummond A. 2012. Geneious basic: an integrated and extendable desktop software platform for the organization and analysis of sequence data. Bioinformatics 28:1647–1649. doi:10.1093/bioinformatics/bts199 22543367 PMC3371832

[38] Li H, Durbin R. 2009. Fast and accurate short read alignment with Burrows-Wheeler transform. Bioinformatics 25:1754–1760. doi:10.1093/bioinformatics/btp324 19451168 PMC2705234

[39] Mirdita M, Steinegger M, Söding J. 2019. MMseqs2 desktop and local web server app for fast, interactive sequence searches. Bioinformatics 35:2856–2858. doi:10.1093/bioinformatics/bty1057 30615063 PMC6691333

[40] Shen W, Ren H. 2021. TaxonKit: a practical and efficient NCBI taxonomy toolkit. J Genet Genomics 48:844–850. doi:10.1016/j.jgg.2021.03.006 34001434

[41] Kumar S, Stecher G, Li M, Knyaz C, Tamura K. 2018. MEGA X: molecular evolutionary genetics analysis across computing platforms. Mol Biol Evol 35:1547–1549. doi:10.1093/molbev/msy096 29722887 PMC5967553

[42] Minh BQ, Schmidt HA, Chernomor O, Schrempf D, Woodhams MD, von Haeseler A, Lanfear R. 2020. IQ-TREE 2: new models and efficient methods for phylogenetic inference in the genomic era. Mol Biol Evol 37:1530–1534. doi:10.1093/molbev/msaa015 32011700 PMC7182206

[43] Martin DP, Murrell B, Golden M, Khoosal A, Muhire B. 2015. RDP4: detection and analysis of recombination patterns in virus genomes. Virus Evol 1:vev003. doi:10.1093/ve/vev003 27774277 PMC5014473

[44] Guo J, Bolduc B, Zayed AA, Varsani A, Dominguez-Huerta G, Delmont TO, Pratama AA, Gazitúa MC, Vik D, Sullivan MB, Roux S. 2021. VirSorter2: a multi-classifier, expert-guided approach to detect diverse DNA and RNA viruses. Microbiome 9:37. doi:10.1186/s40168-020-00990-y 33522966 PMC7852108

[45] Nayfach S, Camargo AP, Schulz F, Eloe-Fadrosh E, Roux S, Kyrpides NC. 2021. CheckV assesses the quality and completeness of metagenome-assembled viral genomes. Nat Biotechnol 39:578–585. doi:10.1038/s41587-020-00774-7 33349699 PMC8116208

[46] Pratama AA, Bolduc B, Zayed AA, Zhong Z-P, Guo J, Vik DR, Gazitúa MC, Wainaina JM, Roux S, Sullivan MB. 2021. Expanding standards in viromics: in silico evaluation of dsDNA viral genome identification, classification, and auxiliary metabolic gene curation. PeerJ 9:e11447. doi:10.7717/peerj.11447 34178438 PMC8210812

[47] Yan M, Pratama AA, Somasundaram S, Li Z, Jiang Y, Sullivan MB, Yu Z. 2023. Interrogating the viral dark matter of the rumen ecosystem with a global virome database. Nat Commun 14:5254. doi:10.1038/s41467-023-41075-2 37644066 PMC10465536

[48] Roux S, Adriaenssens EM, Dutilh BE, Koonin EV, Kropinski AM, Krupovic M, Kuhn JH, Lavigne R, Brister JR, Varsani A, et al.. 2019. Minimum information about an uncultivated virus genome (MIUViG). Nat Biotechnol 37:29–37. doi:10.1038/nbt.4306 30556814 PMC6871006

[49] Bin Jang H, Bolduc B, Zablocki O, Kuhn JH, Roux S, Adriaenssens EM, Brister JR, Kropinski AM, Krupovic M, Lavigne R, Turner D, Sullivan MB. 2019. Taxonomic assignment of uncultivated prokaryotic virus genomes is enabled by gene-sharing networks. Nat Biotechnol 37:632–639. doi:10.1038/s41587-019-0100-8 31061483

[50] Shannon P, Markiel A, Ozier O, Baliga NS, Wang JT, Ramage D, Amin N, Schwikowski B, Ideker T. 2003. Cytoscape: a software environment for integrated models of biomolecular interaction networks. Genome Res 13:2498–2504. doi:10.1101/gr.1239303 14597658 PMC403769

[51] Cantalapiedra CP, Hernández-Plaza A, Letunic I, Bork P, Huerta-Cepas J. 2021. eggNOG-mapper v2: functional annotation, orthology assignments, and domain prediction at the metagenomic scale. Mol Biol Evol 38:5825–5829. doi:10.1093/molbev/msab293 34597405 PMC8662613

[52] Alcock BP, Raphenya AR, Lau TTY, Tsang KK, Bouchard M, Edalatmand A, Huynh W, Nguyen A-LV, Cheng AA, Liu S, et al.. 2020. CARD 2020: antibiotic resistome surveillance with the comprehensive antibiotic resistance database. Nucleic Acids Res 48:D517–D525. doi:10.1093/nar/gkz935 31665441 PMC7145624

[53] Shang J, Peng C, Liao H, Tang X, Sun Y. 2023. PhaBOX: a web server for identifying and characterizing phage contigs in metagenomic data. Bioinform Adv 3:vbad101. doi:10.1093/bioadv/vbad101 37641717 PMC10460485

[54] Krishnan T. 2014. Novel human astroviruses: challenges for developing countries. Virusdisease 25:208–214. doi:10.1007/s13337-014-0202-3 25674587 PMC4188187

[55] De Benedictis P, Schultz-Cherry S, Burnham A, Cattoli G. 2011. Astrovirus infections in humans and animals - molecular biology, genetic diversity, and interspecies transmissions. Infect Genet Evol 11:1529–1544. doi:10.1016/j.meegid.2011.07.024 21843659 PMC7185765

[56] Liu D-Y, Tesh RB, Travassos da Rosa APA, Peters CJ, Yang Z, Guzman H, Xiao S-Y. 2003. Phylogenetic relationships among members of the genus Phlebovirus (Bunyaviridae) based on partial M segment sequence analyses. J Gen Virol 84:465–473. doi:10.1099/vir.0.18765-0 12560581

[57] López Y, Miranda J, Mattar S, Gonzalez M, Rovnak J. 2020. First report of Lihan Tick virus (Phlebovirus, Phenuiviridae) in ticks, Colombia. Virol J 17:63. doi:10.1186/s12985-020-01327-9 32370779 PMC7201772

[58] de Souza WM, Fumagalli MJ, de Araujo J, Ometto T, Modha S, Thomazelli LM, Durigon EL, Murcia PR, Figueiredo LTM. 2019. Discovery of novel astrovirus and calicivirus identified in ruddy turnstones in Brazil. Sci Rep 9:5556. doi:10.1038/s41598-019-42110-3 30944402 PMC6447618

[59] Wang F, Wang M, Dong Y, Zhang B, Zhang D. 2017. Genetic characterization of a novel calicivirus from a goose. Arch Virol 162:2115–2118. doi:10.1007/s00705-017-3302-8 28289976

[60] Mor SK, Phelps NBD, Ng TFF, Subramaniam K, Primus A, Armien AG, McCann R, Puzach C, Waltzek TB, Goyal SM. 2017. Genomic characterization of a novel calicivirus, FHMCV-2012, from baitfish in the USA. Arch Virol 162:3619–3627. doi:10.1007/s00705-017-3519-6 28815386

[61] Purdy MA, Drexler JF, Meng X-J, Norder H, Okamoto H, Van der Poel WHM, Reuter G, de Souza WM, Ulrich RG, Smith DB. 2022. ICTV virus taxonomy profile: Hepeviridae 2022. J Gen Virol 103:36170152. doi:10.1099/jgv.0.001778 PMC12642825 36170152

[62] Kelly AG, Netzler NE, White PA. 2016. Ancient recombination events and the origins of hepatitis E virus. BMC Evol Biol 16:210. doi:10.1186/s12862-016-0785-y 27733122 PMC5062859

[63] Ujike M, Taguchi F. 2021. Recent progress in torovirus molecular biology. Viruses 13:435. doi:10.3390/v13030435 33800523 PMC7998386

[64] Delmas B, Attoui H, Ghosh S, Malik YS, Mundt E, Vakharia VN, ICTV Report Consortium. 2019. ICTV virus taxonomy profile: Picobirnaviridae. J Gen Virol 100:133–134. doi:10.1099/jgv.0.001186 30484763 PMC12662030

[65] Yi Y, Liu S, Hao Y, Sun Q, Lei X, Wang Y, Wang J, Zhang M, Tang S, Tang Q, Zhang Y, Liu X, Wang Y, Xiao X, Jian H. 2023. A systematic analysis of marine lysogens and proviruses. Nat Commun 14:6013. doi:10.1038/s41467-023-41699-4 37758717 PMC10533544

[66] Murphy J, Mahony J, Ainsworth S, Nauta A, van Sinderen D. 2013. Bacteriophage orphan DNA methyltransferases: insights from their bacterial origin, function, and occurrence. Appl Environ Microbiol 79:7547–7555. doi:10.1128/AEM.02229-13 24123737 PMC3837797

[67] Enault F, Briet A, Bouteille L, Roux S, Sullivan MB, Petit MA. 2017. Phages rarely encode antibiotic resistance genes: a cautionary tale for virome analyses. ISME J 11:237–247. doi:10.1038/ismej.2016.90 27326545 PMC5315482

[68] Rasmussen JA, Chua PYS. 2023. Genome-resolving metagenomics reveals wild western capercaillies (Tetrao urogallus) as avian hosts for antibiotic-resistance bacteria and their interactions with the gut-virome community. Microbiol Res 271:127372. doi:10.1016/j.micres.2023.127372 37018898

[69] Koonin EV, Dolja VV, Krupovic M, Varsani A, Wolf YI, Yutin N, Zerbini FM, Kuhn JH. 2020. Global organization and proposed megataxonomy of the virus world. Microbiol Mol Biol Rev 84:e00061-19. doi:10.1128/MMBR.00061-19 32132243 PMC7062200

[70] Kawasaki J, Kojima S, Tomonaga K, Horie M. 2021. Hidden viral sequences in public sequencing data and warning for future emerging diseases. mBio 12:e0163821. doi:10.1128/mBio.01638-21 34399612 PMC8406186

[71] Rappole JH, Hubálek Z. 2003. Migratory birds and West Nile virus. J Appl Microbiol 94 Suppl:47S–58S. doi:10.1046/j.1365-2672.94.s1.6.x 12675936

[72] Tsiodras S, Kelesidis T, Kelesidis I, Bauchinger U, Falagas ME. 2008. Human infections associated with wild birds. J Infect 56:83–98. doi:10.1016/j.jinf.2007.11.001 18096237 PMC7172416

[73] Zhang YZ, Chen YM, Wang W, Qin XC, Holmes EC. 2019. Expanding the RNA virosphere by unbiased metagenomics. Annu Rev Virol 6:119–139. doi:10.1146/annurev-virology-092818-015851 31100994

[74] Yang Q, Rivailler P, Zhu S, Yan D, Xie N, Tang H, Zhang Y, Xu W. 2021. Detection of multiple viruses potentially infecting humans in sewage water from Xinjiang Uygur Autonomous Region, China. Sci Total Environ 754:142322. doi:10.1016/j.scitotenv.2020.142322 33254887

[75] Vlok M, Lang AS, Suttle CA. 2019. Marine RNA virus quasispecies are distributed throughout the oceans. mSphere 4:e00157-19. doi:10.1128/mSphereDirect.00157-19 30944212 PMC6449609

[76] Shi M, Lin X-D, Tian J-H, Chen L-J, Chen X, Li C-X, Qin X-C, Li J, Cao J-P, Eden J-S, Buchmann J, Wang W, Xu J, Holmes EC, Zhang Y-Z. 2016. Redefining the invertebrate RNA virosphere. Nature 540:539–543. doi:10.1038/nature20167 27880757

[77] Cheng R, Li X, Jiang L, Gong L, Geslin C, Shao Z. 2022. Virus diversity and interactions with hosts in deep-sea hydrothermal vents. Microbiome 10:235. doi:10.1186/s40168-022-01441-6 36566239 PMC9789665

[78] Gregory AC, Zablocki O, Zayed AA, Howell A, Bolduc B, Sullivan MB. 2020. The gut virome database reveals age-dependent patterns of virome diversity in the human gut. Cell Host Microbe 28:724–740. doi:10.1016/j.chom.2020.08.003 32841606 PMC7443397

[79] Emerson JB, Roux S, Brum JR, Bolduc B, Woodcroft BJ, Jang HB, Singleton CM, Solden LM, Naas AE, Boyd JA, Hodgkins SB, Wilson RM, Trubl G, Li C, Frolking S, Pope PB, Wrighton KC, Crill PM, Chanton JP, Saleska SR, Tyson GW, Rich VI, Sullivan MB. 2018. Host-linked soil viral ecology along a permafrost thaw gradient. Nat Microbiol 3:870–880. doi:10.1038/s41564-018-0190-y 30013236 PMC6786970

