# Supplementary material for: Highly diverse RNA viruses and phage sequences concealed within birds
Source: Microbiol Spectr. 2024 Jun 11;12(7):e00802-24. doi: 10.1128/spectrum.00802-24 (PMC11218532; doi:10.1128/spectrum.00802-24)

Supplementary Figure 1. The maximum likelihood trees of different RNA viruses were constructed based on RdRp proteins; red branches and black dots indicate novel viruses identified in this study.

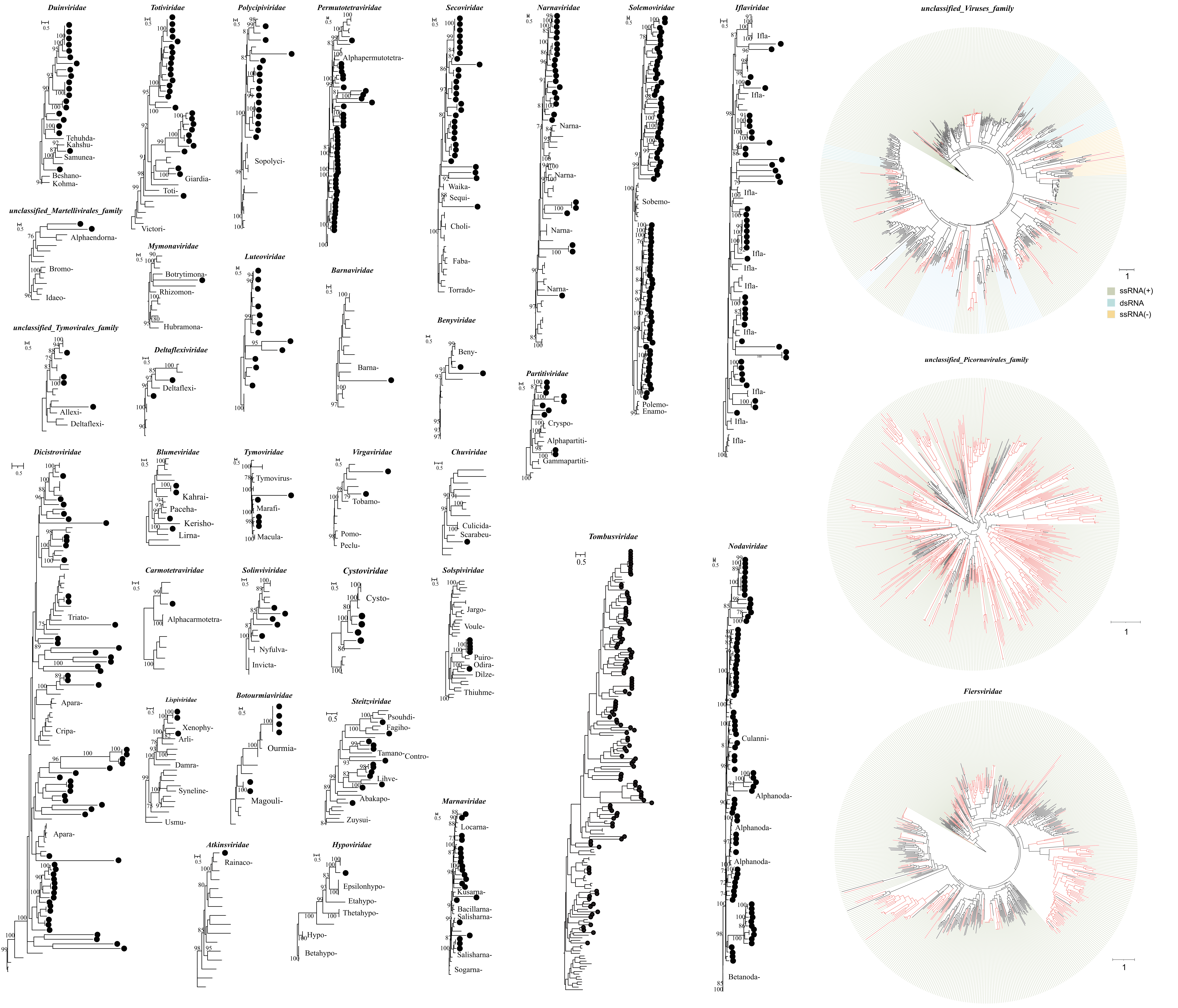

Supplement: Figure S1 — The maximum likelihood trees of different RNA viruses. [file spectrum.00802-24-s0001.pdf]
